# Conversion Therapy of Large Unresectable Hepatocellular Carcinoma With Ipsilateral Portal Vein Tumor Thrombus Using Portal Vein Embolization Plus Transcatheter Arterial Chemoembolization

**DOI:** 10.3389/fonc.2022.923566

**Published:** 2022-06-23

**Authors:** Chengjian He, Naijian Ge, Xiangdong Wang, Hai Li, Shiguang Chen, Yefa Yang

**Affiliations:** ^1^ Mini-Invasive Intervention Center, Eastern Hepatobiliary Surgery Hospital, Second Military Medical University/Navy Medical University, Shanghai, China; ^2^ Department of Interventional Oncology, Fujian Medical University Cancer Hospital, Fuzhou, China

**Keywords:** conversion therapy, initial unresectable, hepatocellular carcinoma (HCC), portal vein embolization (PVE), transcatheter arterial chemoembolization (TACE), tyrosine kinase inhibitors (TKIs)

## Abstract

**Background:**

The study aimed to assess the safety and efficacy of conversion therapy with portal vein embolization (PVE) and transcatheter arterial chemoembolization (TACE) in patients with large unresectable hepatocellular carcinoma (HCC) and ipsilateral portal vein tumor thrombus (PVTT).

**Methods:**

This retrospective study evaluated consecutive patients with initially large (≥5 cm) unresectable HCC with ipsilateral PVTT who underwent PVE + TACE at our center between June 2016 and September 2020 (Group A). Clinically equivalent patients from three centers who were receiving tyrosine kinase inhibitors (TKIs) + TACE (Group B) were included. The survival times were evaluated and compared between the two therapeutic groups.

**Results:**

In Group A (n = 33), the median tumor diameter was 14 cm (range, 5–18 cm) and 19 (57.6%) patients underwent radical resection 18–95 days after PVE. Radical liver resection was not performed because of inadequate hypertrophy (n = 11), pulmonary metastasis (n = 1), lack of consent for surgery (n = 1), and the rupture of the HCC (n = 1). There were no patients who underwent radical resection in Group B (n = 64) (*P* = 0.000). The mean and median overall survival (OS) were 736.5 days and 425.0 days in Group A and 424.5 days and 344.0 days in Group B, respectively. Compared with TKIs + TACE, treatment with PVE + TACE prolonged OS (*P* = 0.023).

**Conclusions:**

This study shows that conversion therapy was safe and effective in patients with initially large unresectable HCC with ipsilateral PVTT treated with PVE + TACE. Moreover, PVE + TACE conferred more favorable outcomes than treatment with TKIs + TACE.

## Introduction

Hepatocellular carcinoma (HCC) is the third cause of cancer-related deaths worldwide and the second cause in China (1, 2). HCC has a strong propensity to invade the adjacent vasculature (3). Portal vein tumor thrombosis (PVTT) is found in 44–62.2% of patients with HCC who have already lost the chance of radical resection; thus, PVTT is recognized as a major prognostic risk factor (4, 5). Even with the best supportive care, the overall survival (OS) of HCC patients with PVTT is only 2–4 months (6, 7). Many factors contribute to the poor prognosis of patients with portal vein invasion, such as more invasive tumor behavior, aggravation of portal hypertension, facilitation of tumor transfer throughout the liver parenchyma or distant metastasis, and decreased hepatopetal portal blood flow (8). The Barcelona Clinic Liver Cancer (BCLC) staging and management system, which is widely accepted and applied in western countries, classifies HCC patients with PVTT as having at least advanced HCC, and systemic therapy with tyrosine kinase inhibitors (TKIs) is recommended as the first-line therapy regardless of PVTT grades (9). Besides TKIs, more aggressive therapies have been used in the clinic, which may improve the prognosis of HCC patients with PVTT and prolong the survival time of patients, such as transarterial chemoembolization (TACE), radiotherapy, hepatic resection, liver transplantation, and various combination of therapies (10–13).

Hepatic resection remains the mainstay of the curative treatment of primary hepatic malignancies. Conversion therapy, which has become a topic of interest for treating advanced liver cancer, converts unresectable advanced HCC or potentially resectable HCC to resectable HCC (14). Conversion therapies for HCC mainly include systemic therapy (15), portal vein embolization (PVE), associating liver partition and portal vein ligation for staged hepatectomy (ALPPS) (16), and TACE. PVE, for the first time reported by Makuuchi et al. (17), is a well-established procedure for inducing future liver remnant (FLR) hypertrophy. The indications for liver resection have been expanded by PVE, which can lower the risk of postoperative liver failure (18).

According to Cheng et al. (11), >90% of PVTT develops on the same side of the lobe of the main tumor, and the tumor thromboses progress in the portal vein with far-from-heart modes. Thus, a combination of PVE and TACE is a potential therapy option for large unresectable HCC with ipsilateral PVTT, in which surgery cannot be performed because of the small FLR and PVTT. This treatment protocol was based on the hypothesis that PVE not only induces FLR hypertrophy but also prevents PVTT from developing toward the main portal vein by mechanically embolizing the targeted portal vein. If a sufficient FLR is achieved and PVTT is successfully treated after PVE, radical surgery can be performed subsequently.

Here, we evaluated 33 patients with initially unresectable large (≥5 cm) HCC with ipsilateral PVTT who underwent PVE and TACE. The oncological results, including OS and progression-free survival (PFS), were compared with those of equivalent patients receiving TKIs + TACE. This study reports our experience with conversion therapy with PVE + TACE in an initially large unresectable HCC with ipsilateral PVTT. Additionally, we intend to evaluate the safety and efficacy of PVE and TACE in these sufferers. To our knowledge, this is the first study that focused on the conversion therapy of large unresectable HCC with ipsilateral PVTT using PVE + TACE.

## Materials and Methods

### Patients

This retrospective study included (a) patients diagnosed with initially large unresectable HCC with ipsilateral PVTT who received PVE + TACE at one center from June 2016 to September 2020, and (b) equivalent patients who received TKIs + TACE at the same center and two other centers. This study was conducted in conformity to the principles of the Declaration of Helsinki. The ethics committee of our hospital authorized the study protocol and waived the need for informed consent.

The criteria for inclusion were as follows: 1) over 18 years of age; 2) histopathologically or radiologically diagnosed HCC on the basis of the American Association for the Study of Liver Diseases criteria; 3) the HCC was unresectable because of PVTT and small standardized FLR (sFLR) (sFLR ≤40% and >30% in patients with cirrhosis, while patients with normal livers generally need ≤30% and >25%); 4) the size of the dominant tumor was ≥5 cm, and the ipsilateral PVTT was of grade Vp1, Vp2, or Vp3 and >1 cm from the main portal vein—according to the Japanese grading system for tumor emboli (19); 5) presence of PVTT spreading from the branches of intrahepatic portal vein (defined as low-attenuation intraluminal filling defect verified by contrast-enhanced computed tomography or magnetic resonance imaging); 6) Child–Pugh class A or B; 7) no extrahepatic invasion; 8) an Eastern Cooperative Oncology Group performance status score of 2 or less; and 9) no contraindication to TACE, PVE, or TKIs. Patients were excluded if they met any of the following criterion: 1) the dominant tumor size of <5 cm or contralateral PVTT; 2) left or right portal vein invasion of <1 cm from the main portal vein or PVTT with Vp4 grade as per the Japanese grading system; 3) other concurrent malignancies; 4) previous therapy for PVTT; 5) received other treatments (including radiofrequency ablation, iodine-125 seed implantation, radiotherapy, etc.) except for the aforementioned treatment for PVTT during the study; 6) sFLR ≤30% in patients with cirrhosis, while sFLR was ≤25% in patients with normal livers, or 7) lost to follow-up.

FLR volume was measured directly by computed tomography and total estimated liver volume (TELV) was calculated from the formula: TELV = −794.41 + 1,267.28 × BSA (body surface, square meters). Then, sFLR was obtained based on the formula: sFLR = FLR/TELV.

Thirty-three patients received PVE + TACE (Group A). Concomitantly, 64 clinically equivalent patients received TKIs + TACE from three centers (Group B).

### Volumetric Assessment

The IQQA-Liver software (EDDA Technology Inc., Princeton, NJ, USA) was used to reconstruct 3-dimensional images and to measure liver volumes. A radical resection operation was considered safe and sufficient when the sFLR ratio was >40% in the cirrhotic liver or >30% in the normal liver.

### TACE

**Figure 1:**
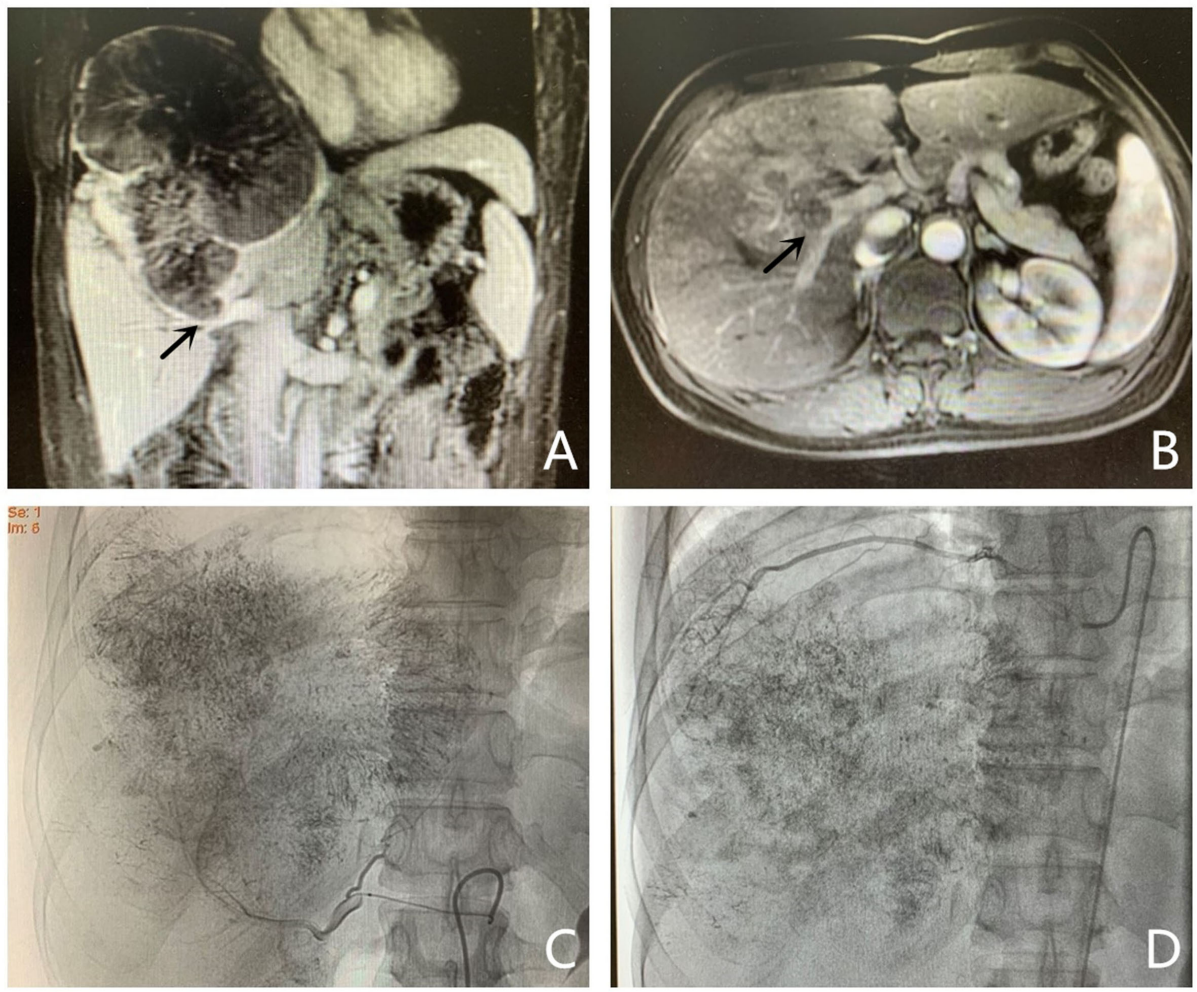
Photographs acquired by magnetic resonance imaging (MRI) and transarterial chemoembolization implemented in a 44-year-old man (PVE + TACE group). **(A, B)** Hepatocellular carcinoma with ipsilateral PVTT, with an intraluminal filling defect which was a low-attenuation and spread from the intrahepatic portal vein branches (black arrow), was detected in the right lobe on enhanced abdominal MRI before therapy. **(C, D)** Images of transarterial chemoembolization. Tumor-feeding arteries are confirmed using digital subtraction angiography. Lipiodol, chemotherapeutic agents, and microspheres were injected into the right hepatic **(C)** and right phrenic arteries **(D)**.

The presence of tumor-feeding arteries was confirmed using digital subtraction angiography. Infusions of Lipiodol (5–20 ml), embolizing fluids, and/or microspheres were infused into the tumor-feeding arteries until the tumor blood flow slowed or stopped completely (**Figure 1**). Patients who underwent TACE were evaluated during follow-up every 4–6 weeks after the procedure. A repeat TACE was performed after confirming Child–Pugh class A or B status, the absence of any liver dysfunction (uncontrolled jaundice, intractable ascites, massive hematemesis, or severe hepatic encephalopathy), and a lesion that was not fully necrotic.

### PVE Procedure

**Figure 2:**
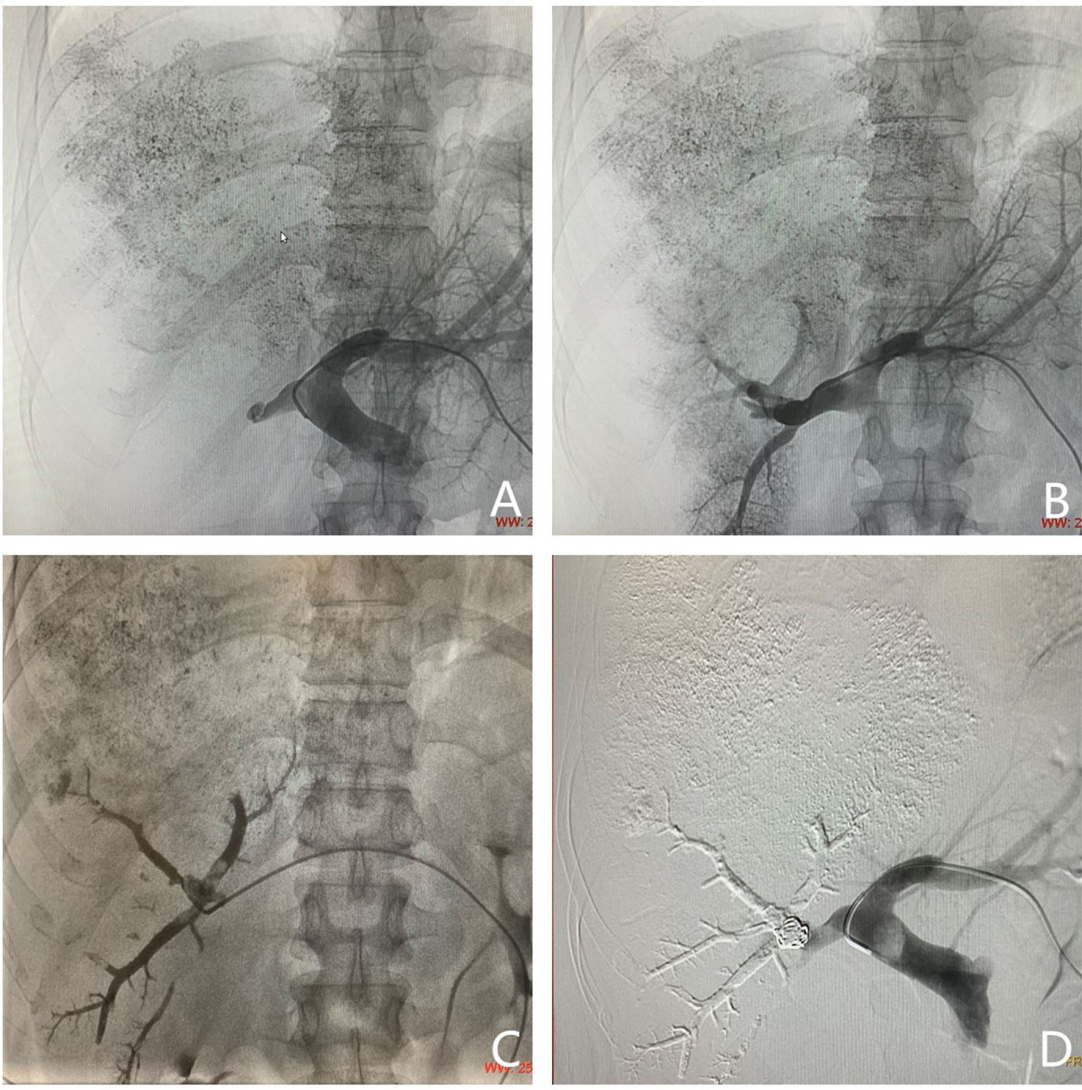
Images of portal vein embolization performed in a 44-year-old man (same patient as in **Figure 1**). Puncturing a suitable branch of the portal vein with the guidance of ultrasound. **(A)** Portography with a catheter placed into the main portal vein. The right portal vein is not completely visible; it appears as a thrombosis in the right portal vein and matched the images acquired by magnetic resonance imaging. **(B)** Portography with a catheter placed into the right portal vein. **(C)** Image showing embolizing of the targeted portal vein. **(D)** Portography after embolizing of the right portal vein. The targeted portal vein was embolized using an NBCA/Lipiodol mixture and coils.

To decrease the risk of PVTT spreading throughout the liver parenchyma or distant metastasis, there are three announcements about PVE for HCC patients with ipsilateral PVTT. First, a contralateral approach is the necessary prerequisite. Second, movements during PVE must be gentle. Third, in the case of migration of PVTT, suction through a catheter should be avoided when the catheter is positioned in the portal vein with PVTT. The contralateral approach provides a more favorable orientation for catheter control and flow-guided distal embolization. Under ultrasound guidance, a suitable branch of the portal vein was punctured by a needle, and portography was performed. The targeted portal vein was embolized using n-butyl cyanoacrylate (NBCA) mixed with iodized oil (n = 19), polyvinyl alcohol (n = 14), gelfoam (n = 6), and/or steel coil (n = 30) (**Figure 2**). All procedures were performed by the same physician. According to a previous report (20), after the embolization, the head end of the catheter was retracted to the hepatic parenchyma. The following day, the catheter was removed.

### Hepatectomy

The tumor was considered resectable if (1) R0 resection could be achieved with sufficient remnant liver volume and function, sFLR >40% in cirrhotic liver and >30% in normal liver, (2) liver function was Child–Pugh stage A or B, (3) there was no extrahepatic metastasis, and (4) no contraindications for hepatectomy existed.

### TKI Therapy and Combination Therapy

TACE combined with TKIs was recommended for patients after assessing its clinical effects, potential adverse events, and costs. Sorafenib, lenvatinib, regorafenib, and apatinib were also recommended. If the recommendation was accepted, TKIs were administered 3–7 days after the first TACE procedure. Sorafenib was administered twice daily, at a dose of 0.4 g. Regorafenib was administered at a dose of 0.16 g once daily. Apatinib was administered at a dose of 0.75 g once daily. Lenvatinib was administered at doses of 8 mg (less than 60 kg) or 12 mg (greater than or equal to 60 kg) once a day. The administration of TKIs was discontinued for three days leading up to the TACE procedure. Therapeutic administration was continued only after any effects of TACE (pyrexia, nausea, or vomiting) subsided. Drug reduction and interruption of drug-related adverse events were permitted.

### Safety Assessment

Adverse events (AEs) were graded based on the Common Terminology Criteria for Adverse Events (version 5.0) and logged during follow-ups at intervals of 1–2 months.

### Follow-Up and Assessment

The outpatient follow-up was conducted every 1–2 months. The censoring date was 5 May 2021. The OS and PFS of the two treatment groups were compared. The definition of OS was the interval from the first TACE session to death or last follow-up. As advanced HCC with PVTT progresses quickly, PVTT progression can induce portal hypertension and deterioration of liver function. The emergence of tumor spread, liver function deterioration, and esophageal and gastric variceal bleeding were indicators of disease progression. The definition of PFS was the interval from the day of the first TACE session to the occurrence of at least one of the aforementioned events or death.

### Statistical Analysis

A Student’s *t*-test was used to compare the continuous variables between the treatment category and baseline characteristics, and Fisher’s exact or χ^2^ test was employed to compare categorical variables. The Kaplan–Meier method was used to estimate survival curves, and the log-rank test was used to analyze differences. Using the Cox regression model, independent prognostic factors, which were correlated with OS identified by univariate analyses, were confirmed through multivariate analyses. SPSS, version 25.0 (SPSS Inc., Chicago, IL, USA), was employed for statistical analyses, with the statistical significance set at P <0.05.

## Results

### Patient Characteristics

**Figure 3:**
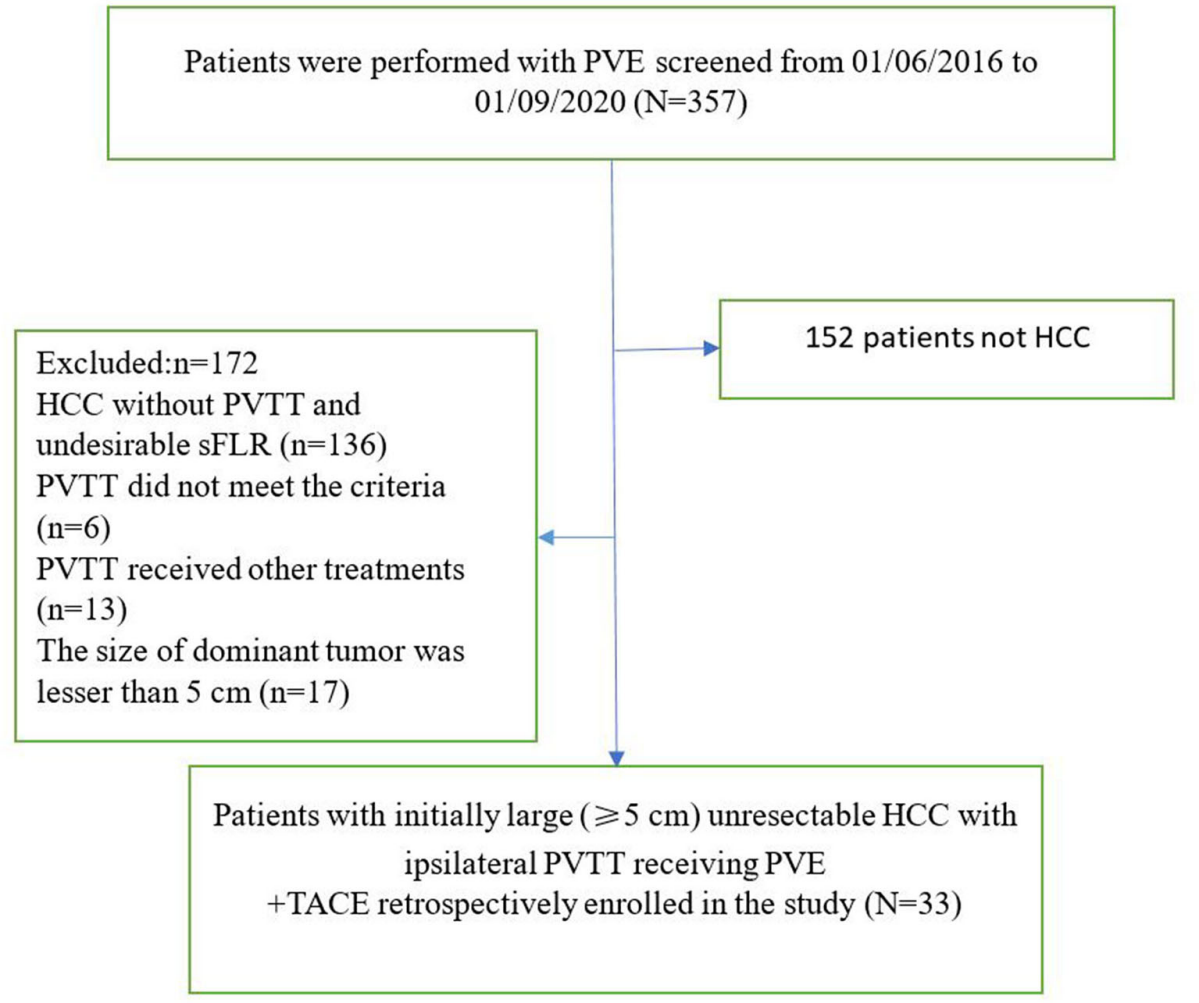
The flow diagram of patients (PVE + TACE group). PVE, portal vein embolization; HCC, hepatocellular carcinoma; PVTT, portal vein tumor thrombus; sFLR, standized future liver remnant; TACE, transarterial chemoembolization.

A total of 357 patients underwent PVE. As shown in **Figure 3**, 152 patients did not have HCC, and 172 patients did not satisfy the enrollment criteria. Thirty-three patients received PVE + TACE (Group A), and 64 patients received TKIs + TACE from three centers (Group B). The baseline characteristics of the two treatment groups are shown in **Table 1**.

**Table 1:** Baseline demographic and clinical characteristics of patients with large unresectable HCC with ipsilateral PVTT.

Characteristics	Group A (N = 33)	Group B (N = 64)	p-value
Age, median (range), years <65 ≥65	51 (43.5–60.5) 29 (87.9) 4 (12.1)	50.5 (43.25–57.75) 59 (92.2) 5 (7.8)	0.982 0.484
Sex (male/female)			0.356
Male	27 (81.8)	57 (89.1)	
Female	6 (18.2)	7 (10.9)	
Child–Pugh			0.157
A	32 (97)	55 (85.9)	
B	1 (3)	9 (14.1)	
Etiology			0.058
Hepatitis B	27 (81.8)	61 (95.3)	
Non-B	6 (18.2)	3 (4.7)	
Tumor number			0.110
Single	26 (78.8)	39 (65.4)	
Multiple	7 (21.2)	25 (34.6)	
HCC maximum diameter (cm)	14 (8.5–15.1)	11.7 (8.3–14.0)	0.138
<10	10 (30.3)	25 (39.1)	0.504
≥10	23 (69.7)	39 (60.9)	
AFP (ng/ml), median (Q1, Q3)	1417 (159.8, 31,114.5)	8,795 (169.2, 112,070.5)	0.289
<400	11 (33.3)	18 (28.1)	0.644
≥400	22 (66.7)	46 (71.9)	
DCP (mAU/ml), median (Q1, Q3)	8,055 (1,745.5, 74,646)	7,664.0 (2,095.0, 29,012)	0.127
<2,050	9 (27.3)	24 (37.5)	0.811
≥2,050	24 (72.7)	48 (62.5)	
PVTT			0.387
VP2	15 (45.5)	23 (69.2)	
VP3	18 (54.5)	41 (30.8)	
TKI			
Sorafenib	0	45	
Lenvatinib	0	15	
Apatinib	0	3	
Regorafenib	0	1	

Data are presented as n (%) or median (Q1, Q3). Q1 and Q3 are 25th percent and 75th percent of interquartile range.

Group A: PVE + TACE group; Group B: TKI +TACE.

PVE, portal vein embolization; TACE, transcatheter arterial chemoembolization; AFP, alpha-fetoprotein concentration; DCP, Des-gammacarboxy prothrombin; TKI, Tyrosine Kinase Inhibitor.

### FLR Hypertrophy and Surgery Rate

**Figure 4:**
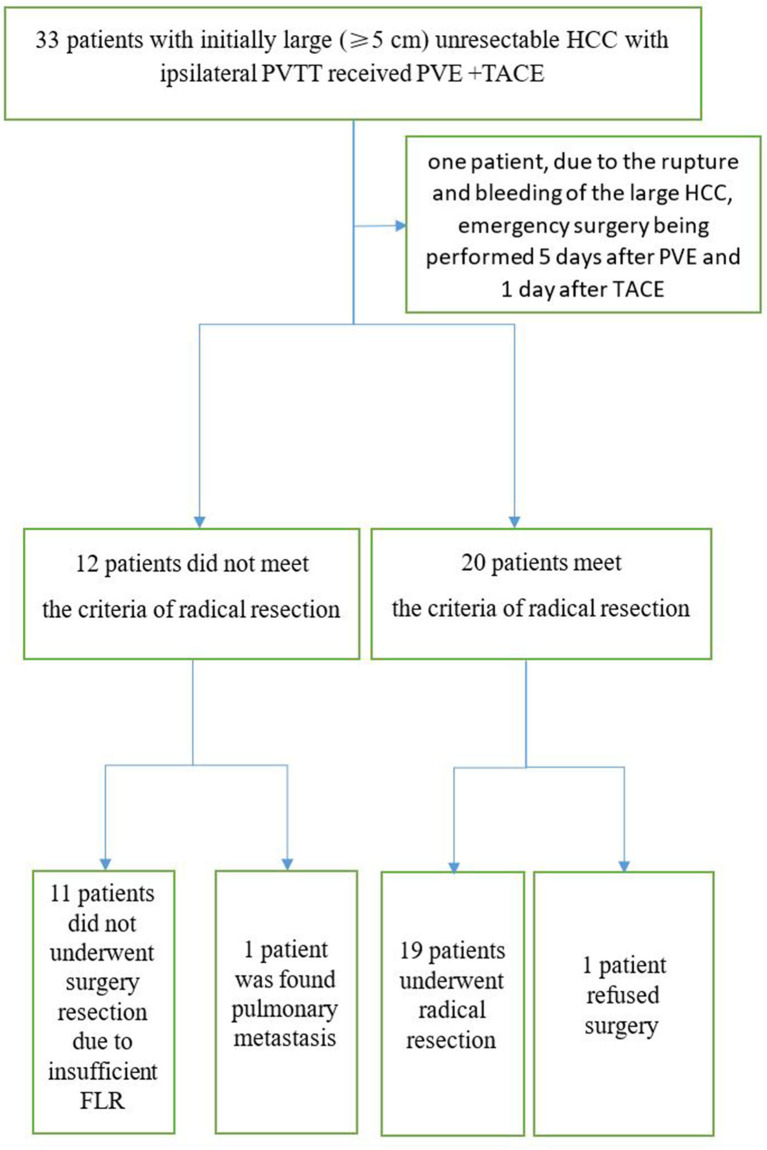
Consolidated Standards of Reporting Trials diagram including all 33 patients who entered the study. PVE, Portal vein emboliaztion; PVTT, Portal vein tumor thrombus; TACE, Transarterial chemoembolization.

**Figure 5:**
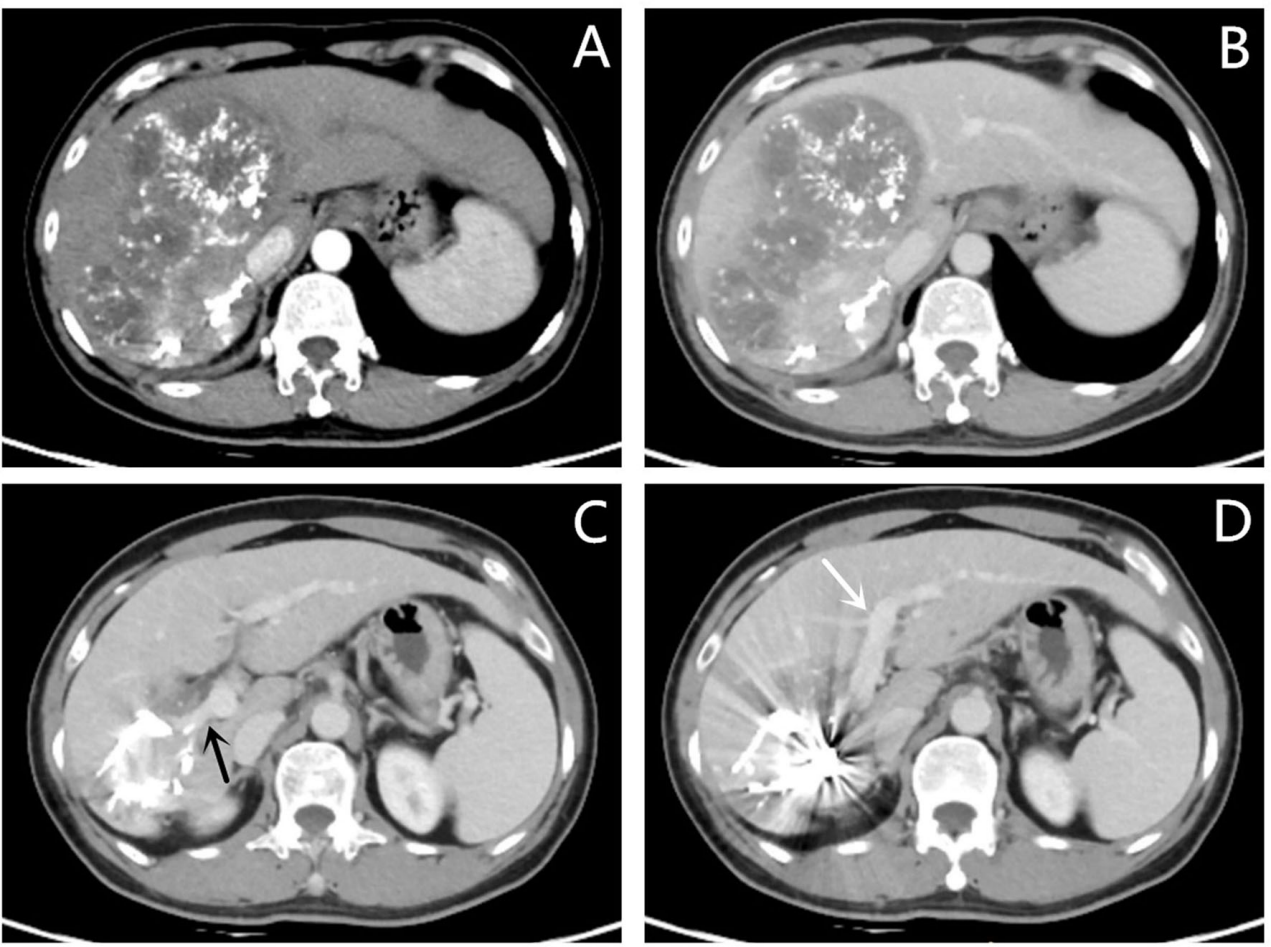
Images of follow-up contrast-enhanced CT of the same patient as in **Figures 1**, **2** after PVE and three sessions of transarterial chemoembolization. **(A, B)** Hepatic arterial phase **(A)** and portal vein phase **(B)** showing that the lesion is fully necrotic. **(C)** Images of the portal vein phase showing a clear initial part of the right portal vein (black arrow). **(D)** Images of the portal vein phase showing a clear left portal vein (white arrow). Compared with **Figure 1**, the left lobe of the liver proliferated significantly after PVE.

In the PVE + TACE group, mean sFLR increased from 29.7% before PVE to 35.9% (*P* =0.000) after PVE, respectively. In Group A, 19 patients (57.6%) underwent radical resection through laparotomy 18–95 days after PVE (**Table 2**). In one patient, because of the rupture and bleeding of the large HCC, an emergency surgery was performed 5 days after PVE and 1 day after the first TACE procedure. Liver resection was not performed because of a small FLR in 11 patients, because of pulmonary metastasis in one patient, and because of lack of consent for surgery in one patient (**Figures 4**, **5**). In the TKIs + TACE group, no patient underwent radical resection (*P* =0.000).

**Table 2:** Characteristics of surgical and postoperative features.

Patient No.	Dominant tumor size, cm	Number of intrahepatic tumors	Japanese grading system	Days after PVE	Disease progression	Alive or die (OS)
1	5	1	Vp2	46	Yes	Die (1281 days)
2	17.3	1	Vp3	23	Yes	Die (677 days)
3	12	1	Vp3	64	Yes	Die (184 days)
4	11.8	1	Vp2	30	Yes	Die (249 days)
5	5.8	2	Vp3	39	Yes	Die (497 days)
6	16.5	1	Vp3	27	Yes	Die (50 days)
7	9	1	Vp2	29	Yes	Die (365 days)
8	12	1	Vp2	22	No	Alive
9	14	1	Vp2	18	Yes	Alive
10	9.3	1	Vp2	95	No	Alive
11	8	1	Vp2	26	No	Alive
12	14	1	Vp3	55	Yes	Alive
13	6.5	1	Vp2	41	Yes	Alive
14	14.7	1	Vp3	55	Yes	Die (200 days)
15	6	2	Vp3	66	Yes	Die (400 days)
16	18	1	Vp3	22	Yes	Alive
17	6	1	Vp3	20	No	Alive
18	7.4	2	Vp2	42	No	Alive
19	10.8	3	Vp1	35	Yes	Alive

PVE, portal vein embolization; OS, overall survival.

### Number of TACE Sessions

Patients in Group A had an average of 2.3 (range, 1–6) TACE sessions and those in Group B had an average of 2.44 sessions (range, 1–13; *P* = 0.70).

### Treatment-related Complications

PVE-related postoperative reactions and TACE-related post-chemoembolization syndrome (pyrexia, nausea, emesis, loss of appetite, and abdominal pain) occurred in nearly all patients. All symptoms are alleviated within a few days of TACE or PVE.

The most common AEs were decreased albumin (16 patients, 48.5%), elevated aspartate transaminase (14 patients, 42.4%), and decreased platelet count (13 patients, 39.4%) in the PVE + TACE group. Grade 3 adverse events included decreased platelet count (two patients, 6.1%), elevated aspartate transaminase level (one patient, 3.0%), and elevated total bilirubin level (one patient, 3.0%). Grade 4 adverse events included rupture and bleeding of the large HCC (1 patient, 3.0%).

### OS Analysis

**Figure 6:**
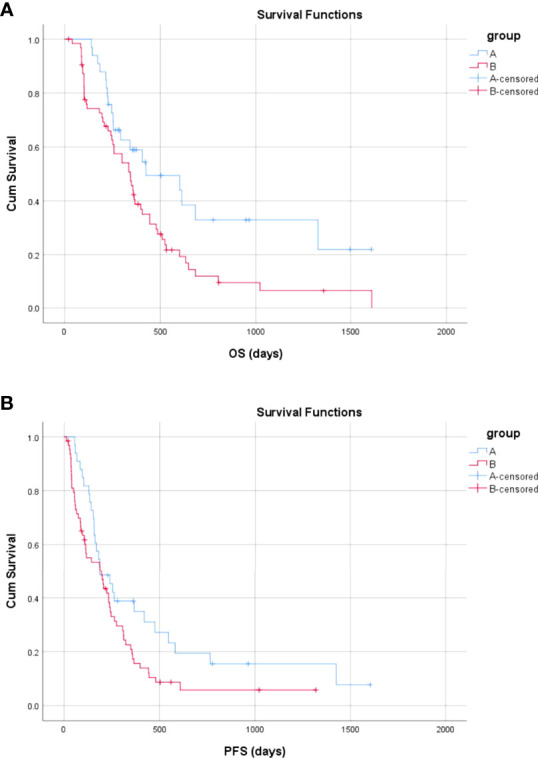
Overall and progression-free survival in Group A (transarterial chemoembolization with portal vein embolization) and Group B (transarterial chemoembolization with molecular targeted therapy). **(A)** The overall survival in Group A and Group B **(B)** The progression-free survival in Group A and Group B.

The median follow-up times in Groups A and B were 777.0 and 805.0 days, respectively (p = 0.220). More patients died in Group B than in Group A during the follow-up period [53 (82.8%) vs. 19 (57.6%); *P* = 0.013]. The mean and median OS were 736.5 days [95% confidence interval (CI) 507.8–965.3 days] and 425.0 days (95% CI, 96.5–753.5 days) in Group A and 424.5 days (95% CI, 319.0–530.1 days) and 344.0 days (95% CI, 251.6–436.4 days) in Group B, respectively. There was a significant difference in OS between Groups B and A (*P* = 0.023) (**Figure 6A**). Compared with TKIs + TACE, the treatment consisting of PVE + TACE prolonged OS.

**Figure 7:**
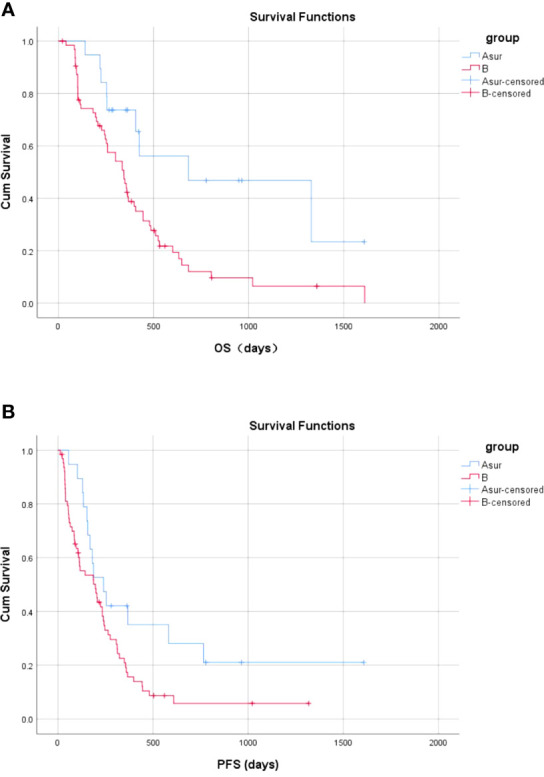
Overall and progression-free survival between Group A_Sur_ and Group B **(A)** The OS in Group A_Sur_ and Group B **(B)** The PFS in the no-operative Group A_Sur_ and Group B.

In Group A, 19 patients underwent radical resection 18–95 days after PVE (Group A_Sur_), and 13 patients did not undergo radical surgery, and one patient underwent emergency surgery 1 day after the first TACE in Group A (Group A_NoSur_). The mean and median OS for patients in Group A_Sur_ were 881.4 days (95% CI, 571.5–1191.3 days) and 684.0 days (95% CI, 0.0–1442.6 days), respectively. There was a significant difference in OS between Group B and Group A_Sur_ (*P* = 0.009) (**Figure 7A**). Compared with TKIs + TACE, treatment consisting of PVE + TACE + sequential radical resection prolonged OS.

**Figure 8:**
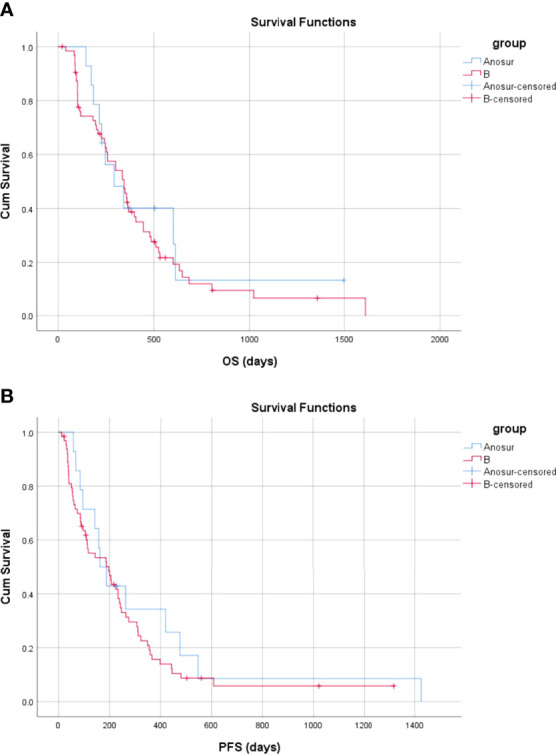
Overall and progression-free survival between Group A_NoSur_ and Group B **(A)** The OS in Group A_NoSur_ and Group B **(B)** The PFS in the no operative Group A_NoSur_ and Group B.

The mean and median OS for patients in Group A_NoSur_ were 501.2 days (95% CI, 239.1–763.2 days) and 292.0 days (95% CI, 131.0–453.0 days), respectively. There was no significant difference in OS between Group B and Group A_NoSur_ (*P* = 0.610) (**Figure 8A**).

### PFS Analysis

The mean and median PFS were 449.6 days (95% CI, 271.8–627.3 days) and 188.0 days (95% CI, 94.8–281.2 days) in Group A and 255.2 days (95% CI, 175.9–334.5 days) and 197.0 days (95% CI, 99.9–294.1 days) in Group B, respectively. There was a significant difference in PFS between Group B and Group A (*P* = 0.047) (**Figure 6B**). Compared with TKIs + TACE, the treatment comprised of PVE + TACE prolonged PFS.

The mean and median PFS were 551.6 days (95% CI, 277.9–825.3 days) and 240.0 days (95% CI, 136.2–343.8 days), respectively, in Group A_Sur_. There was a significant difference in PFS between Group B and Group A_Sur_ (P = 0.037) (**Figure 7B**). Compared with TKIs + TACE, treatment comprised of PVE + TACE + sequential radical resection prolonged PFS.

The mean and median PFS were 336.4 days (95% CI, 124.8–548.1 days) and 162.0 days (95% CI, 105.2–218.8 days) in Group A_NoSur_, respectively. There was no significant difference in PFS between Group B and Group A_NoSur_ (*P* = 0.425) (**Figure 8B**).

### Prognostic Factors for OS

The independent prognostic factors that contributed to OS were confirmed using the Cox regression model. Univariate analysis revealed that OS was correlated with treatment options (*P* = 0.025), maximum tumor diameter <10 cm (*P* = 0.017), and alpha-fetoprotein <400 (*P* = 0.015). Multivariate analysis was performed and maximum tumor diameter <10 cm [HR = 0.538 (95% CI, 0.325–0.890); *P* = 0.016] and treatment options (PVE + TACE) [HR = 0.582 (95% CI, 0.399–1.001); *P* = 0.050] were identified as independent prognostic factors for OS (**Table 3**).

**Table 3:** Univariate and multivariate analysis of predictors associated with overall survival in total cohort.

Variables	n	Univariate Analysis		Multivariate Analysis	
		Hazard Ratio	p-value	Hazard Ratio	p-value
Treatment option PVE + TACE TKI + TACE	33 64	0.547 (0.323–0.928)	0.025	0.582 (0.399–1.001)	0.050
Age, median , years <65 ≥65	88 9	0.839 (0.398–1.767)	0.644		
Sex (male/female)		0.706 (0.349–1.428)	0.333		
Male	13				
Female	84				
Child–Pugh		0.849 (0.598–1.206)	0.380		
A	87				
B	10				
Etiology		1.335 (0.484–3.678)	0.577		
Hepatitis B	88				
Non-B	9				
Tumor number		0.813 (0.501–1.319)	0.402		
Single	65				
Multiple	32				
HCC maximum diameter		0.543 (0.329–0.895)	0.017	0.538 (0.325–0.890)	0.016
<10 cm	35				
≥10 cm	62				
AFP (ng/ml)		0.502 (0.285–0.884)	0.015	0.572 (0.324–1.011)	0.055
<400	28				
≥400	69				
DCP (mAU/ml)		0.806 (0.471–1.380)	0.432		
<2,050	25				
≥2,050	72				
Portal vein invasion grade		1.100 (0.681–1.777)	0.697		
VP2	38				
VP3	59				

PVE, portal vein embolization; TACE, transcatheter arterial chemoembolization; TKI, Tyrosine Kinase Inhibitor; AFP, alpha-fetoprotein concentration; DCP, Des-gammacarboxy prothrombin.

### Subsequent Treatment

Patients with tumor progression underwent subsequent treatment. In Group A, three patients had TKIs and programmed cell death protein-1 (PD-1) inhibitors added to their treatment regimen. In Group B, seven patients had a PD-1 inhibitor added to their primary treatment.

## Discussion

This retrospective study investigated and evaluated the safety and efficacy of conversion therapy with PVE + TACE in patients with an initially large unresectable HCC with ipsilateral PVTT. Additionally, survival time was compared with the equivalent patients from three centers who were receiving TKIs + TACE.

Compared with patients who received TACE with TKIs (Group B), those treated with TACE and PVE (Group A) had a longer OS (736.5 days vs 424.5 days; *P* = 0.023) and PFS (449.6 days vs 255.2 days; *P* = 0.047). In Group A, 19 (57.6%) patients underwent radical resection after PVE, and one patient had an opportunity to undergo radical resection but refused surgery. On evaluation of the group of patients in Group A that did not undergo radical resection (Group A_NoSur_), we found that TACE with PVE in Group A_NoSur_ did not prolong OS (501.2 days vs. 424.5 days; *P* = 0.610) and PFS (336.4 days vs. 255.2 days; *P* = 0.425) compared with TACE and TKIs in Group B. However, compared with TACE and TKIs in Group B, TACE with PVE in Group A_Sur_ prolonged OS (881.4 days vs. 424.5 days; *P* = 0.009) and PFS (551.6 days vs 255.2 days; *P* = 0.037). It can be speculated that radical resection after PVE + TACE in Group A can prolong OS and PFS, but PVE + TACE alone without sequential radical resection cannot prolong OS and PFS compared with TKIs + TACE by mechanically embolizing the targeted portal vein. In other words, the possible reason for this finding was that radical resection, the opportunity for which was provided by PVE through inducing FLR hypertrophy, prolonged OS and PFS. The results suggest that PVE + TACE is a feasible conversion therapy for patients with initially large (>5 cm) unresectable HCC with PVTT to achieve successful resection with a potential long survival time.

Besides surgical resection, targeted therapy (sorafenib and lenvatinib as the first-line treatments; regorafenib, apatinib, cabozantinib, and ramucirumab as the second-line therapies); TACE; radiation therapy; and liver transplantation have been recommended and practiced in HCC patients with PVTT. According to the Asia-Pacific region study, sorafenib monotherapy has only prolonged the survival time for 2–3 months (21). According to Jeong et al. (22), the real-world practical effect of sorafenib monotherapy in HCC with PVTT may be worse because of the selection bias and the median survival time was only 3.1 months. In addition, immunotherapy has developed greatly, and targeted therapy combined with immunotherapy might be a promising treatment strategy. Zhu et al. (15) reported 10 initially unresectable patients (15.9%) underwent R0 resection after TKI and anti-PD-1 antibody combinations, and one of them received PVE due to insufficient FLR. In HCC patients with PVTT, TACE alone had more survival benefit compared to supportive care, which has been confirmed by retrospective and prospective studies (23–25). However, the effect of TACE alone is still unsatisfactory. TACE combined with other therapies may gain a survival benefit and has been recommended as a new therapeutic strategy. With the aid of precision radiotherapy technology, external radiotherapy has been applied to more and more HCC patients with PVTT (26, 27). Compared with external radiotherapy, internal radiotherapy including iodine-125 seed implantation and transarterial radioembolization is more invasive. In comparison with hepatic resection, liver transplantation resolves the lesion and restores liver function simultaneously. Though the indication is expanding, in most research, PVTT is still considered as an absolute contraindication to liver transplantation (28–30).

Hepatic resection is the mainstay of curative treatment for patients with HCC (9). However, resecting HCC with macrovascular invasion is not recommended by the BCLC guidelines (5), while surgical resection is recommended by some Asian guidelines or consensus for patients with PVTT, and highly selected patients meeting these criteria acquire R0 resection (11). A large retrospective study indicated that liver resection could result in survival benefits as long as the PVTT is limited to Vp1–Vp3 (31). However, in this study, before treatment, the tumors were unresectable in all patients because of PVTT and insufficient FLR. Thus, sufficient FLR must be gained before performing curative resection. Besides PVE, ALPPS can also induce FLR hypertrophy (27). In theory, not a surgery-like ALPPS, PVE is an interventional, minimally invasive procedure, which could cause less damage and perioperative complications and is often more acceptable in advanced HCC patients. However, the safety and effectiveness of PVE and ALPPS in large HCC patients with ipsilateral PVTT should be studied by future research.

This study has some limitations. First, selection bias could not be eliminated in this study because of its retrospective character. Second, the number of samples in this study was small. Third, the type of TKI administered was not the same for all patients in Group B. Fourth, the follow-up period was relatively short. Fifth, with the advent of targeted therapy and immunotherapy, conversion therapy for HCC with ipsilateral PVTT in the experimental group excluded TKIs and anti-PD-1 antibodies. Prospective research with a large sample size is essential to verify the efficacy of conversion therapy with PVE, TACE, TKIs, and anti-PD-1 antibodies in HCC patients with ipsilateral PVTT.

Conclusively, the outcome of this study shows that conversion therapy with PVE + TACE could be a safe procedure for patients with large unresectable HCC with ipsilateral PVTT. Besides, the patients who underwent TACE + PVE had longer OS and PFS compared with TACE + TKIs.

## Data Availability Statement

The raw data supporting the conclusions of this article will be made available by the authors, without undue reservation.

## Ethics Statement

The studies involving human participants were reviewed and approved by the Ethics Committee of Eastern Hepatobiliary Surgery Hospital. Written informed consent for participation was not required for this study in accordance with the national legislation and the institutional requirements.

## Author Contributions

CH collected related papers and drafted the manuscript. NG and YY participated in the design of the review. SC was responsible for the supervision of the work. All authors listed have made a substantial, direct, and intellectual contribution to the work and approved it for publication.

## Funding

This work was supported by National Science Foundation of China (No.31971249).

## Conflict of Interest

The authors declare that the research was conducted in the absence of any commercial or financial relationships that could be construed as a potential conflict of interest.

## Publisher’s Note

All claims expressed in this article are solely those of the authors and do not necessarily represent those of their affiliated organizations, or those of the publisher, the editors and the reviewers. Any product that may be evaluated in this article, or claim that may be made by its manufacturer, is not guaranteed or endorsed by the publisher.
